# Supplementary material for: Changes in the behavioural determinants of health during the COVID-19 pandemic: gender, socioeconomic and ethnic inequalities in five British cohort studies
Source: J Epidemiol Community Health. 2021 May 26;75(12):1136–42. doi: 10.1136/jech-2020-215664 (PMC8159672; doi:10.1136/jech-2020-215664)
Supplement: Supplementary data [file jech-2020-215664supp001.pdf]

Before:

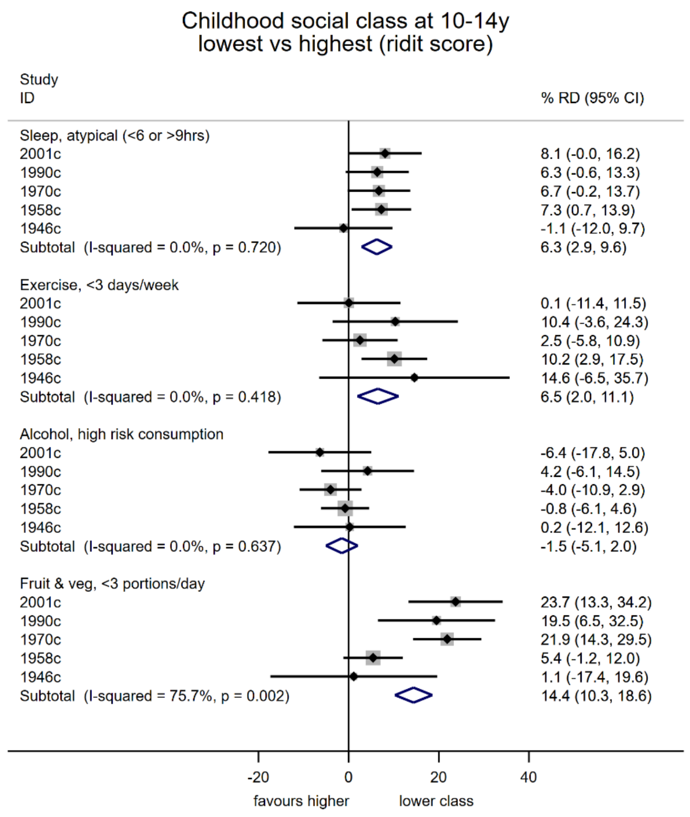

During lockdown:

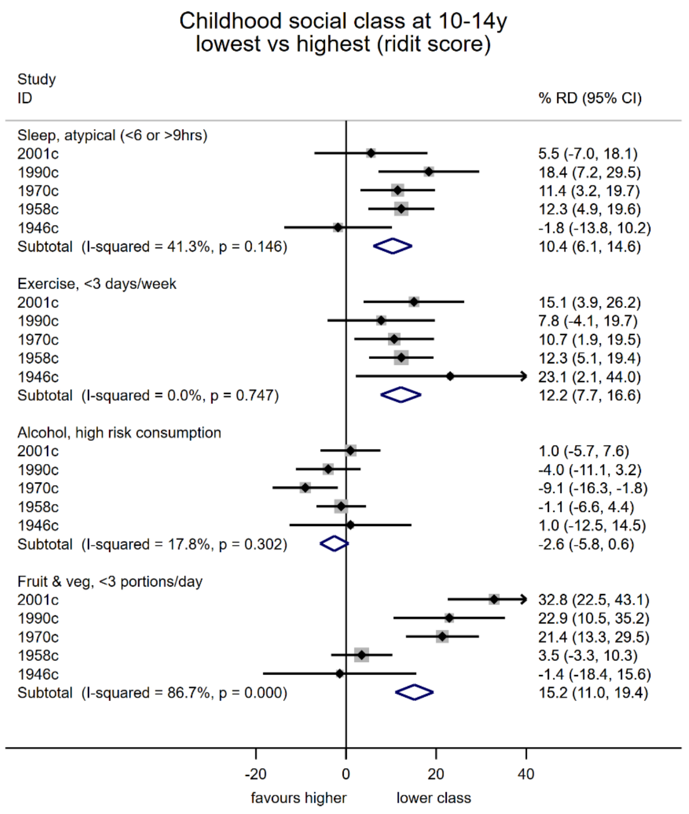

Supplementary Figure 1. Differences in multiple health behaviours during COVID-19 lockdown (May 2020) compared with prior levels, according to gender, education attainment, and ethnicity: a meta-analysis of 5 cohort studies. Note: estimates show the risk difference on the percentage scale and are weighted to account for survey non-response; ridit scores represent the odds ratio of the least compared with most disadvantaged socioeconomic position, the relative index of inequality)

Before:

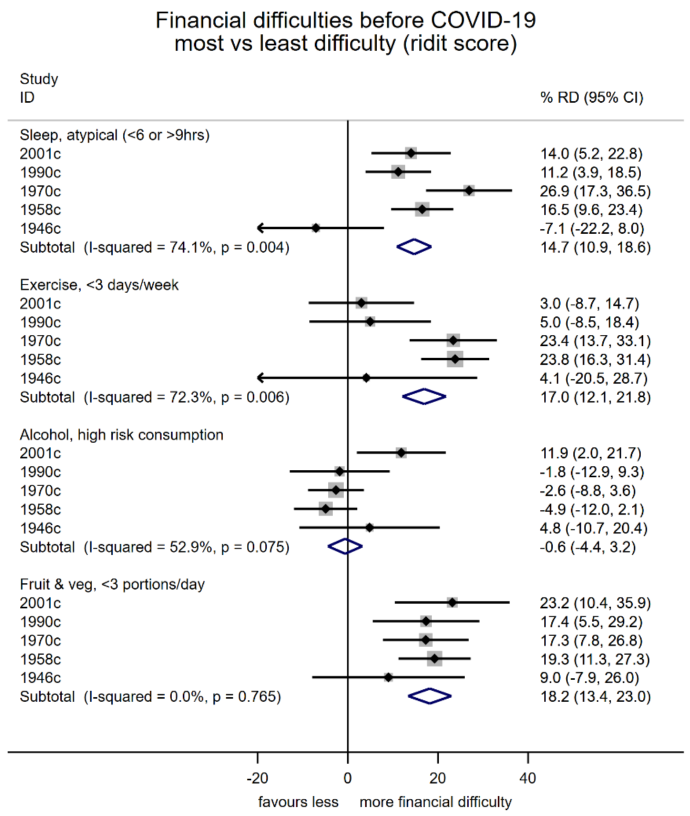

During lockdown:

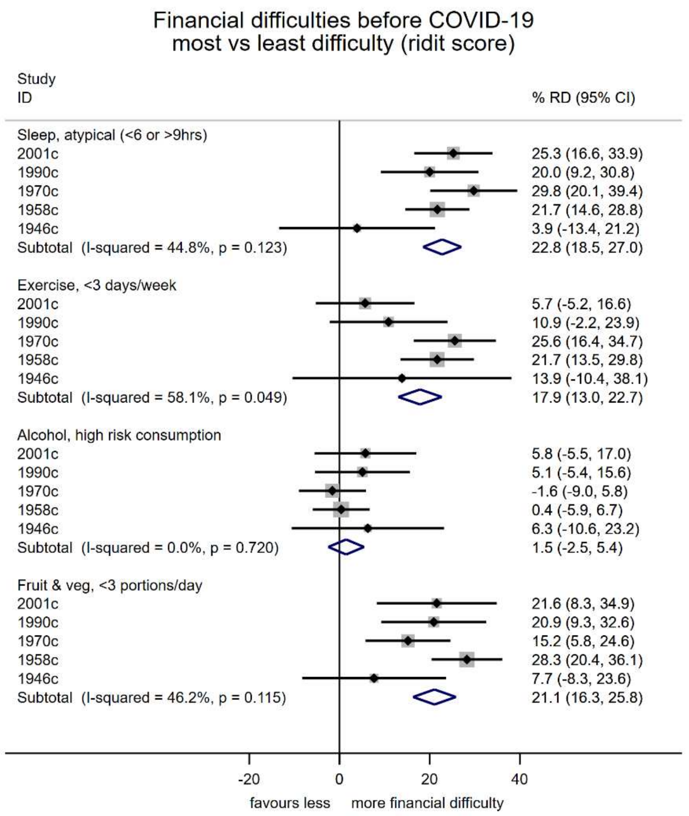

… Supplementary Figure 1 continued.

Supplementary Table 1. Changes in behaviors before and during COVID-19 lockdown: percentages in each cohort and risk factor group.

1) Risk factors according to all cohorts:

	Cohort					Gender		Education attainment		Ethnicity	
**Outcome**, reported change during COVID-19 lockdown	2001	1990	1970	1958	1946	Females	Males	High education (degree/higher)	Low education (GCSE/none)	White	Non-white
**Sleep**											
Less sleep	25.2	21.6	19.5	16.2	13.0	22.5	14.6	17.3	19.7	23.0	28.6
no change	23.0	41.3	53.9	61.2	76.5	48.0	59.3	50.2	57.6	32.0	22.7
More	51.8	37.1	26.5	22.5	10.4	29.5	26.1	32.5	22.7	45.0	48.7
**Exercise**											
Less exercise	33.7	29.5	20.2	18.8	18.1	21.7	23.5	23.2	21.2	30.8	39.8
no change	30.0	35.3	49.1	56.2	59.8	45.7	51.2	42.7	54.3	32.2	32.2
More	36.3	35.2	30.7	25.1	22.1	32.6	25.3	34.2	24.5	37.0	28.1
**Alcohol**											
More alcohol	16.7	30.1	24.4	15.4	10.7	21.4	16.2	20.8	17.3	23.6	13.5
no change	47.5	55.1	67.0	72.7	80.1	63.0	69.9	62.6	68.6	48.7	65.2
Less	35.8	14.8	8.6	11.9	9.2	15.6	13.9	16.6	14.1	27.6	21.3
**Fruit & Veg**											
Less fruit & veg	16.3	16.4	13.9	10.4	5.9	14.5	9.7	12.6	11.3	15.4	22.9
no change	51.7	63.0	70.0	76.6	82.3	66.8	74.2	68.7	73.4	57.7	48.7
More	32.0	20.6	16.2	13.1	11.8	18.7	16.0	18.8	15.3	26.9	28.4

Note: estimates are weighted to account for survey non-response.

2) Risk factors separately in each cohort

Cohort, outcome	reported change during COVID-19 lockdown	Gender: Females	Males	Education attainment: High education (degree/higher)	Low education (GCSE/none)	Ethnicity: White	Non-white
**2001, sleep**	Less sleep	26.2	24.2	21.6	28.5	24.0	34.1
	no change	20.6	25.4	22.9	23.9	24.3	14.5
	More	53.3	50.4	55.5	47.5	51.7	51.4
**2001, exercise**	Less exercise	28.7	38.8	31.7	37.1	32.4	42.6
	no change	28.0	32.0	29.4	29.6	30.3	27.6
	More	43.3	29.2	38.9	33.2	37.3	29.8
**2001, alcohol**	More alcohol	17.0	16.4	16.1	18.2	17.7	9.7
	no change	43.5	51.7	43.2	51.4	45.4	62.9
	Less	39.5	32.0	40.7	30.3	36.9	27.5
**2001, fruit & veg**	Less fruit & veg	16.3	16.4	16.2	15.7	15.0	25.2
	no change	47.4	56.2	48.8	57.6	52.8	42.8
	More	36.3	27.4	35.0	26.7	32.2	32.0
**1990, sleep**	Less sleep	23.2	19.7	18.8	26.4	21.7	21.2
	no change	38.0	45.5	42.3	39.4	42.4	33.7
	More	38.8	34.9	38.9	34.2	35.9	45.1
**1990, exercise**	Less exercise	23.7	36.7	27.3	34.4	28.5	36.0
	no change	38.2	31.8	33.0	38.4	34.9	38.2
	More	38.2	31.5	39.7	27.2	36.6	25.8
**1990, alcohol**	More alcohol	31.7	28.1	30.3	31.1	31.7	18.7
	no change	54.5	56.0	54.8	54.5	53.3	68.4
	Less	13.9	16.0	14.9	14.4	15.1	12.9
**1990, fruit & veg**	Less fruit & veg	18.5	14.0	16.2	13.8	15.9	20.3
	no change	61.7	64.6	63.6	64.4	64.0	55.6
	More	19.8	21.5	20.2	21.9	20.1	24.1

Cohort, outcome	reported change during COVID-19 lockdown	Gender		Education attainment	
		Females	Males	High education (degree/higher)	Low education (GCSE/none)
**1970, sleep**	Less sleep	25.4	13.8	18.1	20.8
	no change	46.2	61.5	51.6	57.1
	More	28.4	24.7	30.3	22.2
**1970, exercise**	Less exercise	20.0	20.5	21.4	18.9
	no change	44.2	53.9	43.7	54.4
	More	35.8	25.7	34.9	26.8
**1970, alcohol**	More alcohol	28.6	20.3	26.3	22.2
	no change	62.5	71.5	65.5	69.2
	Less	8.9	8.2	8.2	8.6
**1970, fruit & veg**	Less fruit & veg	16.8	11.0	14.5	13.6
	no change	64.1	75.6	69.7	72.0
	More	19.0	13.4	15.8	14.4
**1958, sleep**	Less sleep	21.9	10.5	16.1	17.2
	no change	56.0	66.6	60.4	62.5
	More	22.1	23.0	23.5	20.3
**1958, exercise**	Less exercise	18.9	18.6	19.4	16.0
	no change	54.1	58.2	50.4	63.4
	More	27.0	23.2	30.3	20.6
**1958, alcohol**	More alcohol	16.8	14.0	16.8	15.1
	no change	71.1	74.3	72.2	70.4
	Less	12.1	11.8	11.0	14.5
**1958, fruit & veg**	Less fruit & veg	13.2	7.5	9.4	11.1
	no change	73.6	79.5	75.8	77.1
	More	13.2	13.0	14.8	11.8

**Cohort, outcome**	reported change during COVID-19 lockdown	Gender		Education attainment	
		Females	Males	High education (degree/higher)	Low education (GCSE/none)
**1946, sleep**	Less sleep	14.7	11.3	8.9	14.4
	no change	70.1	82.8	75.7	79.0
	More	15.2	5.9	15.4	6.7
**1946, exercise**	Less exercise	21.4	14.9	17.4	15.3
	no change	55.7	63.8	54.9	63.5
	More	22.9	21.4	27.7	21.2
**1946, alcohol**	More alcohol	15.0	6.7	14.1	9.9
	no change	75.0	84.8	72.2	82.2
	Less	10.0	8.5	13.8	7.9
**1946, fruit & veg**	Less fruit & veg	8.4	3.6	6.0	6.0
	no change	80.6	83.9	86.1	82.1
	More	11.1	12.6	7.9	11.9

Supplementary Table 2. Behaviors pre and during COVID-19 lockdown: percentages in each cohort and risk factor group.

		Cohort					Gender		Education		Ethnicity	
**Outcome**	Whole sample	2001	1990	1970	1958	1946	Females	Males	High, degree	Low, GCSE/ none	White	Non-white
**Sleep, pre** (hours/night)												
**1**	0.0%	0.1%	0.0%	0.1%	0.0%	0.0%	0.0%	0.1%	0.0%	0.0%	0.0%	0.0%
**2**	0.1%	0.0%	0.1%	0.1%	0.3%	0.0%	0.2%	0.1%	0.0%	0.2%	0.0%	0.0%
**3**	0.6%	0.5%	0.5%	1.1%	0.4%	0.7%	0.6%	0.6%	0.5%	0.6%	0.5%	0.1%
**4**	1.8%	1.8%	1.3%	2.7%	1.6%	1.0%	2.0%	1.6%	1.3%	1.9%	1.3%	4.2%
**5**	6.2%	4.1%	3.3%	7.3%	6.6%	7.7%	6.5%	5.9%	4.8%	7.8%	3.3%	5.9%
**6**	22.0%	16.3%	20.1%	22.4%	23.7%	25.2%	21.9%	22.2%	19.6%	23.9%	17.7%	20.0%
**7**	33.1%	24.2%	37.7%	37.4%	33.4%	31.2%	32.7%	33.5%	36.7%	29.7%	30.0%	29.8%
**8**	29.2%	34.9%	30.9%	25.1%	28.9%	29.4%	28.5%	29.9%	30.2%	28.8%	34.0%	28.1%
**9**	4.9%	11.7%	4.4%	3.0%	4.0%	3.5%	5.8%	4.1%	5.1%	4.9%	8.8%	6.9%
**10**	1.6%	4.8%	1.3%	0.6%	1.0%	1.3%	1.5%	1.7%	1.4%	1.8%	3.3%	3.4%
**11**	0.2%	0.7%	0.1%	0.0%	0.1%	0.0%	0.2%	0.1%	0.2%	0.1%	0.4%	1.0%
**12**	0.2%	0.7%	0.3%	0.2%	0.1%	0.0%	0.2%	0.2%	0.2%	0.3%	0.5%	0.2%
**13**	0.0%	0.0%	0.0%	0.0%	0.0%	0.0%	0.0%	0.0%	0.0%	0.0%	0.0%	0.0%
**≥14**	0.0%	0.2%	0.0%	0.0%	0.0%	0.0%	0.0%	0.0%	0.0%	0.1%	0.1%	0.4%
**Sleep, during** (hours/night)												
**1**	0.1%	0.3%	0.0%	0.1%	0.2%	0.0%	0.1%	0.1%	0.1%	0.3%	0.2%	0.0%
**2**	0.4%	0.6%	0.3%	0.7%	0.1%	0.1%	0.5%	0.3%	0.2%	0.1%	0.5%	0.1%
**3**	0.8%	0.8%	0.2%	1.2%	0.6%	0.9%	0.8%	0.8%	0.6%	1.1%	0.6%	0.3%
**4**	3.4%	3.1%	2.4%	3.9%	3.6%	3.1%	3.8%	2.9%	2.3%	4.3%	2.5%	5.5%
**5**	7.9%	4.8%	6.3%	9.3%	7.9%	9.6%	8.8%	6.9%	6.3%	9.7%	5.0%	8.3%
**6**	18.1%	10.8%	16.2%	19.2%	19.9%	21.1%	18.5%	17.7%	16.2%	20.1%	12.2%	19.2%
**7**	25.6%	12.1%	23.5%	27.9%	28.0%	32.0%	24.5%	26.8%	26.2%	24.0%	17.8%	11.3%
**8**	27.3%	24.8%	32.3%	26.2%	28.2%	26.4%	26.1%	28.6%	29.8%	25.5%	28.1%	27.3%
**9**	9.9%	20.6%	11.6%	8.2%	8.4%	4.4%	10.2%	9.7%	11.1%	8.8%	17.7%	10.2%
**10**	5.1%	15.6%	5.8%	2.7%	2.9%	2.3%	5.1%	5.0%	5.6%	4.7%	11.5%	10.9%
**11**	0.6%	3.0%	0.3%	0.2%	0.1%	0.0%	0.6%	0.5%	0.6%	0.6%	1.8%	1.7%
**12**	0.7%	3.0%	0.8%	0.2%	0.1%	0.1%	0.9%	0.5%	0.9%	0.6%	1.8%	4.0%
**13**	0.0%	0.2%	0.0%	0.0%	0.0%	0.0%	0.0%	0.0%	0.0%	0.1%	0.1%	0.4%
**≥14**	0.1%	0.4%	0.2%	0.1%	0.0%	0.0%	0.1%	0.1%	0.1%	0.1%	0.3%	0.7%
Sleep, pre *(atypical, <6 or >9)*	10.7%	12.9%	6.9%	12.0%	10.0%	10.8%	11.2%	10.2%	8.5%	12.7%	9.5%	15.2%
Sleep, during *(atypical, <6 or >9)*	19.0%	31.7%	16.5%	18.4%	15.6%	16.1%	20.8%	17.2%	16.7%	21.5%	24.3%	31.9%

**Outcome**	Whole sample	2001	1990	1970	1958	1946	Females	Males	High, degree	Low, GCSE/ none	White	Non-white
**Exercise, pre (times/week)**												
0	19.6%	16.6%	20.2%	19.5%	19.2%	22.7%	22.0%	17.0%	15.6%	23.6%	18.0%	18.5%
1	9.4%	12.2%	11.8%	10.1%	7.7%	6.8%	10.3%	8.4%	10.1%	8.8%	12.4%	9.2%
2	13.8%	15.9%	15.3%	13.4%	13.2%	12.1%	14.5%	13.0%	15.7%	12.9%	15.2%	19.4%
3	15.7%	16.3%	17.8%	16.6%	14.5%	14.5%	17.1%	14.2%	16.6%	14.0%	17.3%	14.6%
4	11.8%	13.0%	10.7%	11.0%	11.2%	13.8%	11.3%	12.4%	11.9%	11.4%	11.6%	15.6%
5	13.6%	12.3%	14.8%	15.2%	14.0%	10.6%	12.1%	15.0%	14.1%	13.3%	13.8%	11.1%
6	4.7%	5.7%	4.1%	3.9%	5.3%	4.5%	3.7%	5.8%	4.8%	4.4%	5.1%	5.3%
7	11.5%	7.8%	5.3%	10.2%	14.9%	15.1%	9.0%	14.1%	11.2%	11.6%	6.7%	6.4%
**Exercise, during (times/week)**												
0	20.7%	16.5%	20.1%	19.9%	21.6%	24.7%	21.6%	19.8%	16.0%	25.1%	17.4%	21.8%
1	9.3%	13.9%	12.4%	9.3%	7.4%	6.0%	9.4%	9.1%	10.1%	8.7%	12.7%	17.3%
2	10.9%	13.0%	10.8%	10.7%	9.8%	11.3%	11.7%	10.1%	10.7%	11.2%	12.4%	10.1%
3	12.9%	13.8%	18.9%	13.9%	10.5%	10.6%	13.5%	12.2%	13.7%	12.2%	16.1%	14.6%
4	10.7%	12.6%	11.5%	10.1%	9.9%	11.0%	10.1%	11.4%	11.4%	9.7%	11.4%	18.0%
5	12.8%	12.1%	10.9%	15.2%	13.4%	10.0%	13.3%	12.4%	13.9%	11.6%	12.1%	8.7%
6	7.0%	7.6%	5.6%	6.4%	8.0%	6.9%	6.9%	7.2%	7.6%	6.1%	7.0%	4.7%
7	15.6%	10.4%	9.8%	14.5%	19.3%	19.5%	13.4%	17.8%	16.7%	15.2%	10.9%	4.9%
Exercise, pre *(<3 times/week)*	28.9%	28.8%	32.0%	29.7%	26.9%	29.5%	32.4%	25.4%	25.7%	32.3%	30.4%	27.7%
Exercise, during *(<3 times/week)*	30.0%	30.4%	32.5%	29.2%	29.1%	30.8%	31.1%	28.9%	26.1%	33.9%	30.1%	39.1%
**Alcohol, pre** (times/week)												
4 or more times a week	18.0%	8.4%	7.3%	15.2%	23.7%	28.5%	13.8%	22.3%	19.7%	16.7%	8.7%	3.0%
2-3 times a week	26.5%	26.2%	19.6%	29.1%	28.2%	24.2%	24.9%	28.0%	30.9%	24.0%	25.0%	13.3%
2-4 times per month	21.4%	29.7%	27.6%	22.2%	17.5%	15.2%	22.2%	20.6%	21.6%	20.4%	30.3%	19.0%
Monthly or less	17.2%	20.4%	25.0%	17.4%	14.4%	13.6%	21.0%	13.3%	14.6%	19.5%	23.0%	16.5%
Never	16.9%	15.3%	20.5%	16.1%	16.2%	18.5%	18.0%	15.8%	13.3%	19.4%	13.0%	48.3%
**Alcohol, during** (times/week)												
4 or more times a week	23.8%	8.9%	14.6%	25.5%	29.2%	32.0%	20.3%	27.4%	26.2%	21.8%	11.8%	8.2%
2-3 times a week	24.2%	19.5%	23.9%	27.0%	25.3%	22.5%	23.4%	25.0%	26.8%	23.1%	23.2%	9.4%
2-4 times per month	17.0%	24.0%	20.4%	16.7%	13.8%	14.2%	17.3%	16.6%	16.5%	16.2%	24.2%	9.3%
Monthly or less	13.0%	19.9%	15.9%	11.9%	10.7%	10.6%	15.3%	10.7%	12.3%	14.0%	18.7%	15.3%
Never	22.0%	27.7%	25.2%	19.0%	20.9%	20.7%	23.7%	20.2%	18.2%	24.8%	22.1%	57.8%
Alcohol, pre *(high risk drinking)*	19.2%	32.6%	16.0%	17.3%	17.2%	14.2%	14.0%	24.3%	21.0%	18.6%	27.4%	13.6%
Alcohol, during *(high risk drinking)*	16.8%	13.0%	12.7%	21.7%	17.4%	14.6%	11.8%	21.9%	18.1%	16.7%	13.3%	10.1%

**Outcome**	Whole sample	2001	1990	1970	1958	1946	Females	Males	High, degree	Low, GCSE/ none	White	Non-white
**Fruit & veg, pre (portions/day)**												
0	1.6%	3.5%	1.5%	1.9%	1.1%	0.3%	1.2%	2.0%	1.0%	1.8%	2.4%	4.3%
1	8.5%	14.0%	8.6%	10.0%	7.4%	3.1%	5.6%	11.4%	7.3%	8.8%	11.4%	13.2%
2	16.3%	21.9%	16.9%	15.5%	15.9%	12.9%	14.7%	18.0%	15.4%	17.0%	19.7%	21.0%
3	23.3%	25.6%	28.4%	22.5%	22.2%	21.2%	23.5%	23.2%	22.8%	24.4%	26.7%	28.5%
4	18.8%	16.9%	16.7%	17.2%	17.8%	26.4%	19.4%	18.2%	19.0%	19.3%	17.5%	10.8%
5	19.4%	10.8%	20.1%	21.7%	21.2%	19.6%	21.1%	17.7%	21.5%	17.7%	15.1%	13.4%
6	12.1%	7.2%	7.8%	11.2%	14.5%	16.5%	14.6%	9.6%	12.9%	11.0%	7.2%	8.8%
**Fruit & veg, during (portions/day)**												
0	1.7%	3.5%	2.5%	2.1%	0.9%	0.2%	1.2%	2.2%	1.4%	2.0%	3.0%	3.3%
1	8.7%	12.1%	9.4%	11.5%	7.5%	3.0%	6.3%	11.2%	7.2%	9.1%	10.2%	16.3%
2	15.0%	18.7%	14.9%	13.8%	16.1%	11.4%	13.8%	16.2%	13.9%	16.1%	17.0%	17.9%
3	22.6%	22.0%	25.0%	23.0%	21.6%	22.9%	23.1%	22.1%	21.1%	24.6%	23.0%	25.3%
4	19.0%	19.4%	19.0%	18.1%	16.8%	24.7%	19.1%	19.0%	19.9%	18.7%	19.7%	15.7%
5	19.2%	14.2%	18.7%	19.6%	21.2%	19.8%	19.9%	18.5%	21.8%	17.6%	16.7%	12.2%
6	13.7%	10.0%	10.4%	12.0%	15.9%	18.0%	16.6%	10.8%	14.8%	12.1%	10.3%	9.2%
Fruit & veg, pre *(< 3 portions a day)*	26.4%	39.4%	27.0%	27.4%	24.3%	16.3%	21.4%	31.3%	23.8%	27.6%	33.5%	38.5%
Fruit & veg, during *(< 3 portions a day)*	25.4%	34.4%	26.9%	27.3%	24.5%	14.7%	21.3%	29.6%	22.5%	27.1%	30.3%	37.5%

Notes:
High risk drinking is consuming more than 14 drinks a week or more than 5 drinks in a typical drinking day.
Measures in italics are those used in the main analyses shown in Figure 2.
